# Electrocaloric Effect in Different Oriented BaZr_0.15_Ti_0.85_O_3_ Single Crystals

**DOI:** 10.3390/ma15197018

**Published:** 2022-10-10

**Authors:** Yun Ou, Chihou Lei, Dongliang Shan

**Affiliations:** 1 School of Materials Science and Engineering, Hunan University of Science and Technology, Xiangtan 411201, China; 2 Department of Aerospace and Mechanical Engineering, Saint Louis University, Saint Louis, MI 63103-1110, USA; 3 Key Laboratory of Low Dimensional Materials and Application Technology of Ministry of Education, School of Materials Science and Engineering, Xiangtan University, Xiangtan 411105, China

**Keywords:** electrocaloric effect, barium zirconate titanate, oriented single crystal, broad work temperature range

## Abstract

The electrocaloric effect of ferroelectrics is promising for new solid-state refrigeration. However, the current research on the electrocaloric effect of bulk ferroelectrics mainly focuses on (001) orientation. Thus, we studied the electrocaloric effect of BaZr_0.15_Ti_0.85_O_3_ single crystals with different orientations through the nonlinear thermodynamic approach and entropy analysis. The results show that the dipolar entropy of (111)-oriented BaZr_0.15_Ti_0.85_O_3_ single crystals exhibits a greater change after applying an external electric field, compared with (001)- and (110)-orientations, and the (001)-oriented electrocaloric responses are consistent with experimental observations. The (111)-oriented BaZr_0.15_Ti_0.85_O_3_ single crystals have a more significant electrocaloric response, resulting in a broader work temperature range with a large electrocaloric effect. These insights offer an alternative way to enhance the electrocaloric response of ferroelectric single crystals.

## 1. Introduction

The solid-state refrigeration technology based on the electrocaloric (EC) effect of ferroelectric materials has the characteristics of low noise, high efficiency, and being environmentally friendly [1,2,3,4,5,6,7,8]. It is expected to become a new generation of refrigeration ways leading to potential application prospects [9,10,11]. Existing studies have shown that the large electrocaloric effect of ferroelectric materials usually occurs near the Curie temperature [12,13,14,15,16]. For example, Mischenko et al. discovered a giant electrocaloric effect of about 12 K under an electric field of 48 MV/m near the Curie temperature in PbZr_0.95_Ti_0.05_O_3_ thin film [12], while Neese et al. discovered a temperature change of 12 K under an electric field of 209 MV/m around the Curie temperature in polymer thin films [17]. However, thin films are limited in practical EC cooling applications due to their smaller thermal mass than bulks [3]. Therefore, the ferroelectric single crystal bulk material has attracted attention. For example, Moya et al. found an electrocaloric effect temperature change of 0.9 K under an electric field of 1.2 MV/m near the Curie temperature in BaTiO_3_ single crystal through experiments [13], while Wu et al. found maximum EC strength near the phase boundary of PbMg_1/3_Nb_2/3_O_3_-PbTiO_3_ single crystals by molecular dynamics theory [18].

In recent years, lead-free barium zirconate titanate (BaZr_x_Ti_1–x_O_3_) has attracted much attention due to its rich phase structure and excellent electrocaloric (EC) properties [19,20,21,22,23,24]. Direct measurement by Qian et al. indicates that when the change of the electric field is 2.1 MV/m, BaZr_0.2_Ti_0.8_O_3_ ceramics at 38 °C exhibit an EC temperature change of 1.1 °C with a large EC coefficient of around 0.5 × 10^−6^ KmV^−1^ [19]. Compared with other ferroelectric bulk materials, it possesses better EC properties and is an EC material with great application potential [19,20].

Ferroelectric materials with different orientations generate different phase structures, which affect the corresponding material properties [25,26]. Xu et al., using a thermodynamic model, studied the orientation-dependent equilibrium ferroelectric domain structures and dielectric properties of polydomain PbZr_1-*x*_Ti*_x_*O_3_ thin films; the film orientation can deeply influence the phase stability and properties [25]. Li et al. studied the orientation-related electrocaloric effect of 0.7Pb(Mg_1/3_Nb_2/3_)O_3_-0.3PbTiO_3_ (PMN-0.3PT) single crystals, and the (111) oriented PMN-0.3PT single crystals showed a larger electrocaloric response than (110) and (111) orientations near Curie temperature under the electric field of 2.5 kV/cm [27]. The electrocaloric effect is greatly affected by the crystal orientations. However, the current research on BaZr_x_Ti_1–x_O_3_ and other ferroelectric single crystals mainly focuses on the (001) orientation, while the other orientations, such as the common (110) and (111) orientations, are rarely reported. Therefore, in this letter, the polarization and phase structures of BaZr_0.15_Ti_0.85_O_3_ in (001), (110), and (111) orientations are established by thermodynamic methods. The resulting entropies and the EC temperature changes under different electric fields are further computed. Comparing the EC temperature changes for the three orientations will provide a guideline for enhancing the electrocaloric effect of ferroelectric single crystals.

## 2. Theoretical Approach

The thermodynamic potential energy of bulk ferroelectrics under stress-free boundary conditions can be expressed as a polynomial of the polarization component $P_{l}^{\prime }\left(l=1,2,3\right)$ and electric field component $E_{l}^{\prime }$ [28,29,30,31,32,33,34,35],

(1) $$\begin{array}{c} G^{\prime }\left(E,T\right)=\alpha_{1}\left({P_{1}^{\prime }}^{2}+{P_{2}^{\prime }}^{2}+{P_{3}^{\prime }}^{2}\right)+\alpha_{11}\left({P_{1}^{\prime }}^{4}+{P_{2}^{\prime }}^{4}+{P_{3}^{\prime }}^{4}\right)+\alpha_{12}\left({P_{1}^{\prime }}^{2}{P_{2}^{\prime }}^{2}+{P_{2}^{\prime }}^{2}{P_{3}^{\prime }}^{2}+{P_{1}^{\prime }}^{2}{P_{3}^{\prime }}^{2}\right)+\\ \alpha_{111}\left({P_{1}^{\prime }}^{6}+{P_{2}^{\prime }}^{6}+{P_{3}^{\prime }}^{6}\right)+\alpha_{112}\left[{P_{1}^{\prime }}^{2}\left({P_{2}^{\prime }}^{4}+{P_{3}^{\prime }}^{4}\right)+{P_{2}^{\prime }}^{2}\left({P_{1}^{\prime }}^{4}+{P_{3}^{\prime }}^{4}\right)+{P_{3}^{\prime }}^{2}({P_{1}^{\prime }}^{4}+{P_{2}^{\prime }}^{4})\right]+\alpha_{123}{P_{1}^{\prime }}^{2}{P_{2}^{\prime }}^{2}{P_{3}^{\prime }}^{2}+\\ \alpha_{1111}\left({P_{1}^{\prime }}^{8}+{P_{2}^{\prime }}^{8}+{P_{3}^{\prime }}^{8}\right)+\alpha_{1122}\left({P_{1}^{\prime }}^{4}{P_{2}^{\prime }}^{4}+{P_{2}^{\prime }}^{4}{P_{3}^{\prime }}^{4}+{P_{1}^{\prime }}^{4}{P_{3}^{\prime }}^{4}\right)+\alpha_{1112}[{P_{1}^{\prime }}^{6}\left({P_{2}^{\prime }}^{2}+{P_{3}^{\prime }}^{2}\right)+{P_{2}^{\prime }}^{6}\left({P_{1}^{\prime }}^{2}+{P_{3}^{\prime }}^{2}\right)+\\ {P_{3}^{\prime }}^{6}({P_{1}^{\prime }}^{2}+{P_{2}^{\prime }}^{2})]+\alpha_{1123}\left({P_{1}^{\prime }}^{4}{P_{2}^{\prime }}^{2}{P_{3}^{\prime }}^{2}+{P_{1}^{\prime }}^{2}{P_{2}^{\prime }}^{4}{P_{3}^{\prime }}^{2}+{P_{1}^{\prime }}^{2}{P_{2}^{\prime }}^{2}{P_{3}^{\prime }}^{4}\right)-P_{1}^{\prime }E_{1}^{\prime }-P_{2}^{\prime }E_{2}^{\prime }-P_{2}^{\prime }E_{2}^{\prime } \end{array}$$

where $\alpha_{1}$, $\alpha_{ij}$, $\alpha_{ijk}$, and $\alpha_{ijkl}$ are the thermodynamic potential coefficients. Note that all the coefficients are independent of *T* except $\alpha_{1}$, $\alpha_{1}=\left(T-T_{0}\right)/2\varepsilon_{0}C$, which satisfies the Curie–Weiss law, *T*_0_ is the Curie–Weiss temperature, *C* is the Curie constant, and ε_0_ is the permittivity of vacuum. 

In this work, three typical orientations, (001), (110), and (111), are considered. The crystallographic axes ($x_{1}^{\prime }x_{2}^{\prime }x_{3}^{\prime }$) denote the local coordinate system, where $x_{1}^{\prime }$||[100], $x_{2}^{\prime }$||[010], $x_{3}^{\prime }$||[001]. For (001)-, (110)-, and (111)-oriented bulk ferroelectrics, the transformation matrices from local coordinates to global coordinates are expressed as

(2) $$T_{\left(001\right)}=\left[\begin{array}{ccc} 1 & 0 & 0\\ 0 & 1 & 0\\ 0 & 0 & 1 \end{array}\right],T_{\left(110\right)}=\left[\begin{array}{ccc} 0 & 0 & 1\\ \frac{1}{\sqrt{2}} & -\frac{1}{\sqrt{2}} & 0\\ \frac{1}{\sqrt{2}} & \frac{1}{\sqrt{2}} & 0 \end{array}\right],\;T_{\left(111\right)}=\left[\begin{array}{ccc} \frac{1}{\sqrt{2}} & -\frac{1}{\sqrt{2}} & 0\\ \frac{1}{\sqrt{6}} & -\frac{1}{\sqrt{6}} & -\frac{2}{\sqrt{6}}\\ \frac{1}{\sqrt{3}} & \frac{1}{\sqrt{3}} & \frac{1}{\sqrt{3}} \end{array}\right]$$

The polarization $P^{\prime }$ and the electric field $E^{\prime }$, with respect to the local coordinates, are related to their corresponding global physical quantities, that is, $P^{\prime }=T_{\left(hkl\right)}^{-1}P$ and $E^{\prime }=T_{\left(hkl\right)}^{-1}E$, where **P** and **E** are the physical quantities with respect to the global coordinates. Notice that the superscript “−1” refers to the matrix inverse, and the subscript (*hkl*) represents the involved orientation (001), (110), or (111). Thus, the thermodynamic potential energy density of bulk ferroelectric with the (001), (110), and (111) orientations, denoted by *G*_(001)_, *G*_(110)_, and *G*_(111)_, are obtained and presented in the Supplementary Materials, as Equations (S1)–(S3).

To predict the temperature change of the electrocaloric effect of the ferroelectric single crystals with different orientations, the total entropy $S_{total}$ of the ferroelectric crystals is decomposed according to $S_{total}\left(E,T\right)=S_{dip}\left(E,T\right)+S_{latt}\left(T\right)$ [36,37], where $S_{dip}\left(E,T\right)$ is the dipolar entropy related to the polarization **P**, and $S_{latt}\left(T\right)$ is the lattice entropy, which is independent of the applied electric field E. $S_{latt}\left(T\right)$ is assumed to be only correlated to lattice contribution. Due to the adiabatic condition, the total entropy change of the system is zero. The initial state entropy ($E_{i},T_{i}$), equal to the final state entropy ($E_{f},T_{f}$), leads to

(3) $$S_{latt}\left(T_{f}\right)-S_{latt}\left(T_{i}\right)=-[S_{dip}\left(E_{f},T_{f}\right)-S_{dip}\left(E_{i},T_{i}\right)],$$

Moreover, the change of lattice entropy can be approximated by

(4) $$S_{latt}\left(T_{f}\right)-S_{latt}\left(T_{i}\right)=\int_{T_{i}}^{T_{f}}\frac{C_{latt}\left(T\right)}{T}dT\approx C_{latt}\left(T_{i}\right)\ln\left(\frac{T_{f}}{T_{i}}\right),\;$$

The approximation is employed in Equation (4) because the variation of $C_{latt}\left(T\right)$ on the interval [*T_i_*, *T_f_*] is insignificant. The temperature at the final state $T_{f}$ can be determined as [36,37,38]

(5) $$T_{f}=T_{i}exp\left\{-\frac{1}{C_{latt}}\left[S_{dip}\left(E_{f},T_{f}\right)-S_{dip}\left(E_{i},T_{i}\right)\right]\right\}.$$

The adiabatic temperature change of the electrocaloric effect can be computed

(6) $$\Delta T=T_{f}-T_{i}=T_{i}exp\left\{-\frac{1}{C_{latt}}\left[S_{dip}\left(E_{f},T_{f}\right)-S_{dip}\left(E_{i},T_{i}\right)\right]\right\}-T_{i},$$

where $C_{latt}$ is the lattice heat capacity per unit volume, the subscript i indicates the initial state, and f indicates the final state, revealing that the adiabatic temperature change is connected with the dipolar entropy $S_{dip}\left(E,T\right)=-{\left[\partial G\left(E,T\right)/\partial T\right]}_{E}$ [38]. Notice that both sides of Equation (6) involve initial temperature $T_{i}$ and final temperature $T_{f}$, and thus are solved numerically by iteration. The polarization can be determined by solving the equilibrium equation ${\left(\partial G/\partial P_{l}\right)}_{E}=0$ [39]. The lattice heat capacity is acquired from available experimental data [40]. For the sake of convenience, the electric field is assumed to be directed along [001] direction in the global coordinate, and the initial electric field $E_{i}$ is chosen as zero. The electrocaloric calculations were carried out at temperatures from 20 °C to 140 °C, and the polar axis, favorable to ferroelectricity, is along the (111) direction.

## 3. Results and Discussion

**Figure 1:**
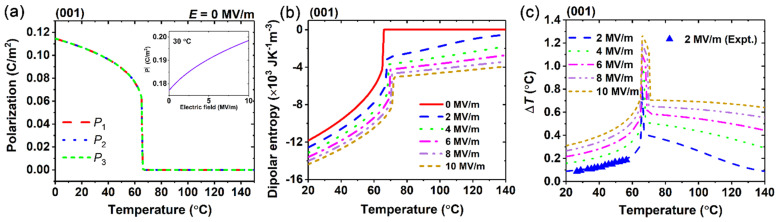
(**a**) Polarizations of (001)-oriented BaZr_0.15_Ti_0.85_O_3_ in the absence of an external electric field, and modulus of polarization vector as electric field increases at 30 °C. (**b**) Dipolar entropy and (**c**) adiabatic temperature change Δ*T* under different electric fields as a function of temperature. Experimental data are denoted by blue triangles [21].

We first investigated the EC effect in the common (001)-oriented BaZr_0.15_Ti_0.85_O_3_ single crystal, as shown in Figure 1. In the absence of an applied electric field, the polarization is shown in Figure 1a, and its Curie temperature is around 66 °C, which is good agreement with experimental observations [41,42]. The polarizations below the Curie temperature appear as *P*_1_ = *P*_2_ = *P*_3_ ≠ 0, representing the R phase, and the modulus of the polarization vector increases with the electric field increase, which predicts that the dipoles are more ordered under the electric field, further affecting the dipole entropy. Figure 1b demonstrates the trend of dipolar entropy over a temperature range under different electric fields. The dipolar entropy increases with increasing temperature but decreases with an increasing electric field. A more significant change in the dipolar entropy is observed near the Curie temperature, indicating a larger electrocaloric effect near the Curie temperature.

The EC temperature change of the (001)-oriented BaZr_0.15_Ti_0.85_O_3_ single crystal over a range of operating temperature, as shown in Figure 1c, increases until reaching the largest value at the Curie temperature. As the electric field increases, the range of the large EC temperature change becomes wider, due to the increase of the phase transition temperature. For instance, when the electric field change is 2 MV/m, the computational and the experimental data are consistent [21]. Moreover, when the electric field is changed to 10 MV/m, the maximum EC temperature change of the (001)-oriented BaZr_0.15_Ti_0.85_O_3_ single crystal is 1.27 °C.

**Figure 2:**
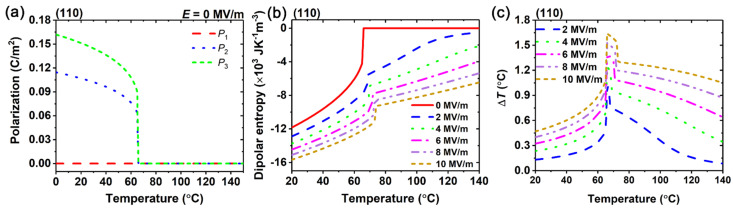
(**a**) Polarizations of (110)-oriented BaZr_0.15_Ti_0.85_O_3_ in the absence of an external electric field. (**b**) Dipolar entropy and (**c**) adiabatic temperature change Δ*T* under different electric fields as a function of temperature.

The EC effect of the (110)-oriented BaZr_0.15_Ti_0.85_O_3_ single crystals is illustrated in Figure 2. Below the Curie temperature, the polarizations appear as *P*_1_ = 0, *P*_2_ ≠ 0, and *P*_3_ ≠ 0, is shown in Figure 2a. Compared with the (001) orientation, the dipolar entropy of the BaZr_0.15_Ti_0.85_O_3_ single crystals in the (110) orientation experience a greater change after the electric field is applied, as shown in Figure 2b. Thus, the EC temperature change of (110)-oriented crystals can be enhanced from the (001)-oriented crystals. For instance, when the electric field is changed to 10 MV/m, the maximum EC temperature change is 1.64 °C.

**Figure 3:**
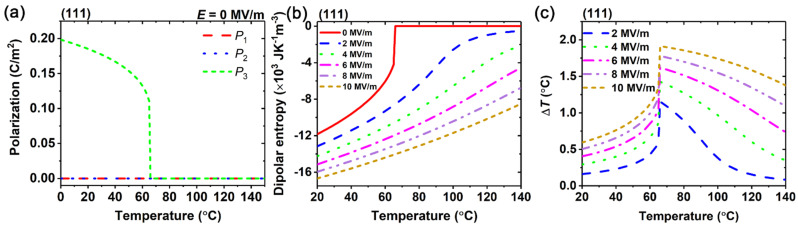
(**a**) Polarizations of (111)-oriented BaZr_0.15_Ti_0.85_O_3_ in the absence of external electric field. (**b**) Dipolar entropy and (**c**) adiabatic temperature change Δ*T* under different electric fields as a function of temperature.

The trends of polarization, entropy, and the electrocaloric effect of (111)-oriented BaZr_0.15_Ti_0.85_O_3_ single crystals are shown in Figure 3. The trend of polarization below the Curie temperature, with *P*_1_ = *P*_2_ =0 and *P*_3_ ≠ 0, is shown in Figure 3a. Compared with the dipolar entropies of the (001)- and (110)-oriented BaZr_0.15_Ti_0.85_O_3_ single crystals, the dipolar entropy of the (111)-oriented BaZr_0.15_Ti_0.85_O_3_ single crystals under an external electric field varies continuously across the Curie temperature, as shown in Figure 3b. Thus, the (111)-orientated BaZr_0.15_Ti_0.85_O_3_ single crystals possess a broader temperature range for the large electrocaloric temperature change. For instance, when the electric field is changed at 10 MV/m, the maximum EC temperature change can reach 1.91 °C, while the EC temperature change is larger than 1.5 °C within the temperature range from 66 °C to 128 °C.

**Figure 4:**
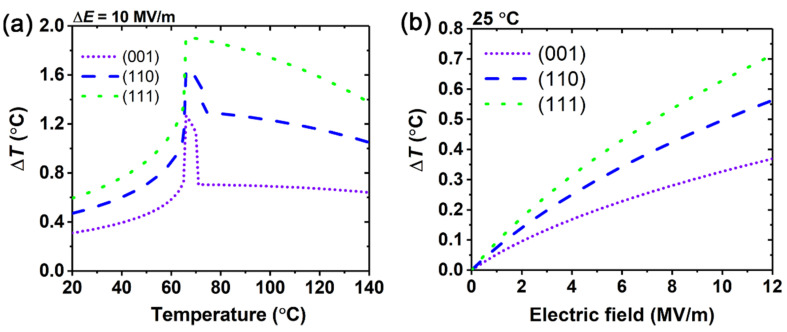
Comparison of electrocaloric responses in BaZr_0.15_Ti_0.85_O_3_ single crystals with different orientations. (**a**) As a function of temperature under an electric field change Δ*E* = 10 MV/m; (**b**) As a function of an electric field at 25 °C.

Finally, we compare the electrocaloric effects of the three orientations, as shown in Figure 4. The EC temperature changes of BaZr_0.15_Ti_0.85_O_3_ single crystals in the three orientations are shown in Figure 4a when the electric field is changed to 10 MV/m. The maximum EC temperature change of the three orientations occurs at 66 °C. The maximum EC temperature change of the (111)-oriented crystals is about 1.5 times that of the (001)-oriented crystal, indicating that the electrocaloric effect becomes significantly enhanced. It can be seen from Figure 4a that the (111)-oriented crystals exhibit a wider temperature range with a large EC temperature response. The trend of the EC temperature changes of BaZr_0.15_Ti_0.85_O_3_ single crystals with the three orientations at 25 °C over the electric field are shown in Figure 4b. With the increase of the electric field, the EC temperature changes of the three orientations all increase, where those of (110)- and (111)-orientations are significantly higher than those of the (001)-orientation. The EC temperature change of the (111)-orientation is about 1.9 times that of the (001)-orientation at 12 MV/m. Due to the external applied electric field direction being the same as the polarization direction in (111)-oriented BaZr_0.15_Ti_0.85_O_3_ single crystals, the polarization of (111)-oriented BaZr_0.15_Ti_0.85_O_3_ single crystals is easier to change compared with (001)- and (110)-orientations, as the more polarization change lead to more entropy change, resulting in a larger electrocaloric effect temperature change in (111)-oriented BaZr_0.15_Ti_0.85_O_3_ single crystals. This suggests that the EC performance of the ferroelectric single crystals can be enhanced by choosing suitable orientations.

## 4. Conclusions

In conclusion, the polarization, the dipole entropy, and the electrocaloric temperature changes of (001)-, (110)-, and (111)-oriented BaZr_0.15_Ti_0.85_O_3_ single crystals were studied by developing the thermodynamic theory. The predicted results are in good agreement with the experiments. Compared to the (001)- and (110)-orientations, the polarization of the (111)-oriented BaZr_0.15_Ti_0.85_O_3_ single crystals is greatly improved, where the dipolar entropy further increases after the electric field is applied. Thus, (111)-oriented BaZr_0.15_Ti_0.85_O_3_ single crystals exhibit excellent electrocaloric performance with a broader work temperature range of large EC temperature changes, which provides insight into enhancing the EC properties of ferroelectric single crystals in general.

## Data Availability

Not applicable.

## Supplementary Materials

The following supporting information can be downloaded at: https://www.mdpi.com/article/10.3390/ma15197018/s1, Equation (S1): The thermodynamic potential energy density of the (001)-oriented bulk ferroelectric, Equation (S2): The thermodynamic potential energy density of the (110)-oriented bulk ferroelectric, Equation (S3): The thermodynamic potential energy density of the (111)-oriented bulk ferroelectric.

## References

[1] Wang Y., Zhang Z., Usui T., Benedict M., Hirose S., Lee J., Kalb J., Schwartz D. (2020). A high-performance solid-state electrocaloric cooling system. Science.

[2] Qian X.S., Han D.L., Zheng L.R., Chen J., Tyagi M., Li Q., Du F.H., Zheng S., Huang X.Y., Zhang S.H. (2021). High-entropy polymer produces a giant electrocaloric effect at low fields. Nature.

[3] Shi J.Y., Han D.L., Li Z.C., Yang L., Lu S.G., Zhong Z.F., Chen J.P., Zhang Q.M., Qian X.S. (2019). Electrocaloric cooling materials and devices for zero-global-warming-potential, high-efficiency refrigeration. Joule.

[4] Li X.Y., Lu S.G., Chen X.Z., Gu H.M., Qian X.S., Zhang Q.M. (2013). Pyroelectric and electrocaloric materials. J. Mater. Chem. C.

[5] Scott J.F. (2011). Electrocaloric materials. Annu. Rev. Mater. Res..

[6] Shi J., Zhu R.F., Liu X., Fang B.J., Yuan N.Y., Ding J.N., Luo H.S. (2017). Large electrocaloric effect in lead-free (Ba_0.85_Ca_0.15_)(Zr_0.1_Ti_0.9_)O_3_ ceramics prepared via citrate route. Materials.

[7] Asbani B., Gagou Y., Ben Moumen S., Dellis J.-L., Lahmar A., Amjoud M.B., Mezzane D., El Marssi M., Rozic B., Kutnjak Z. (2022). Large electrocaloric responsivity and energy storage response in the lead-free Ba(Ge_x_Ti_1−x_)O_3_ ceramics. Materials.

[8] Hou X., Li H.Y., Shimada T., Kitamura T., Wang J. (2018). Effect of geometric configuration on the electrocaloric properties of nanoscale ferroelectric materials. J. Appl. Phys..

[9] Correia T., Zhang Q. (2014). Electrocaloric Materials: New Generation of Coolers.

[10] Moya X., Mathur N.D. (2020). Caloric materials for cooling and heating. Science.

[11] Meng Y., Zhang Z.Y., Wu H.X., Wu R.Y., Wu J.H., Wang H.L., Pei Q.B. (2020). A cascade electrocaloric cooling device for large temperature lift. Nat. Energy.

[12] Mischenko A.S., Zhang Q., Scott J.F., Whatmore R.W., Mathur N.D. (2006). Giant electrocaloric effect in thin-film PbZr_0.95_Ti_0.05_O_3_. Science.

[13] Moya X., Stern-Taulats E., Crossley S., Gonzalez-Alonso D., Kar-Narayan S., Planes A., Manosa L., Mathur N.D. (2013). Giant electrocaloric strength in single-crystal BaTiO_3_. Adv. Mater..

[14] Shan D.L., Pan K., Lei C.H., Peng J.L., He N.B., Pan J.Y., Jin H.Y., Liu Y.Y. (2019). Large electrocaloric response over a broad temperature range near room temperature in Ba_x_Sr_1__−x_TiO_3_ single crystals. J. Appl. Phys..

[15] Moya X., Kar-Narayan S., Mathur N.D. (2014). Caloric materials near ferroic phase transitions. Nat. Mater..

[16] Bai G., Qin X.S., Xie Q.Y., Gao C.F. (2019). Electric-field-induced phase transition and electrocaloric effect in PZT near morphotropic phase boundary. Phys. B.

[17] Neese B., Chu B.J., Lu S.G., Wang Y., Furman E., Zhang Q.M. (2008). Large electrocaloric effect in ferroelectric polymers near room temperature. Science.

[18] Wu H.H., Cohen R.E. (2017). Electric-field-induced phase transition and electrocaloric effect in PMN-PT. Phys. Rev. B.

[19] Qian X.S., Ye H.J., Zhang Y.T., Gu H.M., Li X.Y., Randall C.A., Zhang Q.M. (2014). Giant electrocaloric response over a broad temperature range in modified BaTiO_3_ ceramics. Adv. Funct. Mater..

[20] Qian J.F., Hu P.H., Liu C., Jiang J.Y., Dan Z.K., Ma J., Lin Y.H., Nan C.W., Shen Y. (2018). High electrocaloric cooling power of relaxor ferroelectric BaZr*_x_*Ti_1–*x*_O_3_ ceramics within broad temperature range. Sci. Bull..

[21] Jian X.D., Lu B., Li D.D., Yao Y.B., Tao T., Liang B., Guo J.H., Zeng Y.J., Chen J.L., Lu S.G. (2018). Direct measurement of large electrocaloric effect in Ba(Zr_x_Ti_1−x_)O_3_ ceramics. ACS Appl. Mater. Inter..

[22] Shan D.L., Cai Y.C., Lei C.H., Peng J.L., He N.B., Pan K., Liu Y.Y., Li J.Y. (2021). Electric-field-driven coexistence of positive and negative electrocaloric effects near room temperature for high-efficiency two-stage cooling. Appl. Phys. Lett..

[23] Peng J.L., Shan D.L., Liu Y.Y., Pan K., Lei C.H., He N.B., Zhang Z.Y., Yang Q. (2018). A thermodynamic potential for barium zirconate titanate solid solutions. npj Comput. Mater..

[24] Co K., Khassaf H., Alpay S.P. (2020). Electrocaloric and pyroelectric properties of barium zirconate titanate. J. Appl. Phys..

[25] Xu R.J., Zhang J.L., Chen Z.H., Martin L.W. (2015). Orientation-dependent structural phase diagrams and dielectric properties of PbZr_1__−x_Ti_x_O_3_ polydomain thin films. Phys. Rev. B.

[26] Xu R.J., Liu S., Grinberg I., Karthik J., Damodaran A.R., Rappe A.M., Martin L.W. (2015). Ferroelectric polarization reversal via successive ferroelastic transitions. Nat. Mater..

[27] Li J.T., Yin R.W., Su X.P., Wu H.H., Li J.J., Qin S.Q., Sun S.D., Chen J., Su Y.J., Qiao L.J. (2020). Complex phase transitions and associated electrocaloric effects in different oriented PMN-30PT single crystals under multi-fields of electric field and temperature. Acta Mater..

[28] Liu Y.Y., Zhu Z.X., Li J.F., Li J.Y. (2010). Misfit strain modulated phase structures of epitaxial Pb(Zr_1__−x_Ti_x_)O_3_ thin films: The effect of substrate and film thickness. Mech. Mater..

[29] Liu Y.Y., Li J.Y. (2011). Shear-driven morphotropic phase boundary in epitaxial ferroelectric thin films. Phys. Rev. B.

[30] Liu Y.Y., Yang L., Li J.Y. (2013). Strain-engineered orthorhombic-rhombohedral phase boundary in epitaxial bismuth ferrite films. J. Appl. Phys..

[31] Li Y.L., Cross L.E., Chen L.Q. (2005). A phenomenological thermodynamic potential for BaTiO_3_ single crystals. J. Appl. Phys..

[32] Liu Y.Y., Vasudevan R.K., Pan K., Xie S.H., Liang W.I., Kumar A., Jesse S., Chen Y.C., Chu Y.H., Nagarajan V. (2012). Controlling magnetoelectric coupling by nanoscale phase transformation in strain engineered bismuth ferrite. Nanoscale.

[33] Liu N., Su Y., Weng G.J. (2013). A phase-field study on the hysteresis behaviors and domain patterns of nanocrystalline ferroelectric polycrystals. J. Appl. Phys..

[34] Pertsev N.A., Zembilgotov A.G., Tagantsev A.K. (1998). Effect of mechanical boundary conditions on phase diagrams of epitaxial ferroelectric thin films. Phys. Rev. Lett..

[35] Liu Y.Y., Li J.Y. (2019). Seeing is believing: Negative capacitance captured at both nano- and macro-scales. Sci. Bull..

[36] Pirc R., Kutnjak Z., Blinc R., Zhang Q.M. (2011). Electrocaloric effect in relaxor ferroelectrics. J. Appl. Phys..

[37] Liu Y., Scott J.F., Dkhil B. (2016). Direct and indirect measurements on electrocaloric effect: Recent developments and perspectives. Appl. Phys. Rev..

[38] Shan D.L., Lei C.H., Cai Y.C., Pan K., Liu Y.Y. (2021). Mechanical control of electrocaloric response in epitaxial ferroelectric thin films. Int. J. Solids Struct..

[39] Peng J.L., Li Q., Shan D.L., Pan K., Yu G.S., Liu Y.Y. (2016). Phenomenological thermodynamic potentials for bulk and thin-film Ba(Zr_0.08_Ti_0.92_)O_3_ single crystals. J. Appl. Phys..

[40] Jian X.D., Lu B., Li D.D., Yao Y.B., Tao T., Liang B., Lu S.G. (2018). Large electrocaloric effect in lead-free Ba(Zr_x_Ti_1−x_)O_3_ thick film ceramics. J. Alloys Compd..

[41] Mahesh M.L.V., Prasad V.V.B., James A.R. (2014). Enhanced dielectric and ferroelectric properties of lead-free Ba(Zr_0.15_Ti_0.85_)O_3_ ceramics compacted by cold isostatic pressing. J. Alloys Compd..

[42] Yu Z., Guo R.Y., Bhalla A.S. (2000). Dielectric behavior of Ba(Ti_1__−x_Zr_x_)O_3_ single crystals. J. Appl. Phys..

