# Supplementary material for: Electrocaloric Effect in Different Oriented BaZr0.15Ti0.85O3 Single Crystals
Source: Materials (Basel). 2022 Oct 10;15(19):7018. doi: 10.3390/ma15197018 (PMC9572230; doi:10.3390/ma15197018)
Supplement: Supplementary file 1 [file materials-15-07018-s001.zip › materials-1904593-supplementary.pdf]

SUPPLEMENTARY DATA

Electrocaloric effect in different oriented $BaZr_{0.15}Ti_{0.85}O_3$ single crystals

Yun Ou[1], Chihou Lei[2], and Dongliang Shan[3,*]

1. School of Materials Science and Engineering, Hunan University of Science and Technology, Xiangtan, Hunan 411201, China
2. Department of Aerospace and Mechanical Engineering, Saint Louis University, Saint Louis, Missouri 63103-1110, USA
3. Key Laboratory of Low Dimensional Materials and Application Technology of Ministry of Education, School of Materials Science and Engineering, Xiangtan University, Xiangtan, Hunan 411105, China

The thermodynamic potential energy density of the (001)-oriented bulk ferroelectric is expressed as:

$$G_{(001)}(\mathbf{E},T)=\alpha_1(P_1^2+P_2^2+P_3^2)+\alpha_{11}(P_1^4+P_2^4+P_3^4)+\alpha_{12}(P_1^2P_2^2+P_2^2P_3^2+P_1^2P_3^2)+\alpha_{111}(P_1^6+P_2^6+P_3^6)+\alpha_{112}[P_1^2(P_2^4+P_3^4)+P_2^2(P_1^4+P_3^4)+P_3^2(P_1^4+P_2^4)]+\alpha_{123}P_1^2P_2^2P_3^2+\alpha_{1111}(P_1^8+P_2^8+P_3^8)+\alpha_{1122}(P_1^4P_2^4+P_2^4P_3^4+P_1^4P_3^4)+\alpha_{1112}[P_1^6(P_2^2+P_3^2)+P_2^6(P_1^2+P_3^2)+P_3^6(P_1^2+P_2^2)]+\alpha_{1123}(P_1^4P_2^2P_3^2+P_1^2P_2^4P_3^2+P_1^2P_2^2P_3^4)-P_1E_1-P_2E_2-P_3E_3, \quad \text{(S1)}$$

The thermodynamic potential energy density of the (110)-oriented bulk ferroelectric is expressed as:

$$G_{(110)}(\mathbf{E},T)=\alpha_1(P_1^2+P_2^2+P_3^2)+\frac{1}{2}\alpha_{11}(2P_1^4+P_2^4+6P_2^2P_3^2+P_3^4)+\frac{1}{4}\alpha_{12}(4P_1^2P_2^2+P_2^4+4P_1^2P_3^2-2P_2^2P_3^2+P_3^4)+\frac{1}{4}\alpha_{111}(4P_1^6+P_2^6+15P_2^4P_3^2+15P_2^2P_3^4+P_3^6)+\frac{1}{4}\alpha_{112}(4P_1^4P_2^2+2P_1^2P_2^4+P_2^6+4P_1^4P_3^2+12P_1^2P_2^2P_3^2-P_2^4P_3^2+2P_1^2P_3^4-P_2^2P_3^4+P_3^6)+\frac{1}{4}\alpha_{123}(P_1^2P_2^4-2P_1^2P_2^2P_3^2+P_1^2P_3^4)+\frac{1}{16}[\alpha_{1111}(16P_1^8+2P_2^8+56P_2^6P_3^2+140P_2^4P_3^4+56P_2^2P_3^6+2P_3^8)+\alpha_{1112}(16P_1^6P_2^2+4P_1^2P_2^6+2P_2^8+16P_1^6P_3^2+60P_1^2P_2^4P_3^2+8P_2^6P_3^2+60P_1^2P_2^2P_3^4-20P_2^4P_3^4+4P_1^2P_3^6+8P_2^2P_3^6+2P_3^8)+\alpha_{1122}(8P_1^4P_2^4+$$

* Authors to whom the correspondence should be addressed to: dlshan@xtu.edu.cn

$P_2^8 + 48P_1^4P_2^2P_3^2 - 4P_2^6P_3^2 + 8P_1^4P_3^4 + 6P_2^4P_3^4 - 4P_2^2P_3^6 + P_3^8) + \alpha_{1123}(4P_1^4P_2^4 + 4P_1^2P_2^6 - 8P_1^4P_2^2P_3^2 - 4P_1^2P_2^4P_3^2 + 4P_1^4P_3^4 - 4P_1^2P_2^2P_3^4 + 4P_1^2P_3^6)] - P_1E_1 - P_2E_2 - P_3E_3,$ (S2)

The thermodynamic potential energy density of the (111)-oriented bulk ferroelectric is expressed as:

$$G_{(111)}(\mathbf{E},T) = \alpha_1(P_1^2 + P_2^2 + P_3^2) + \frac{1}{6}\alpha_{11}\big(3P_1^4 + 6P_1^2P_2^2 + 3P_2^4 + 12\sqrt{2}P_1^2P_2P_3 - 4\sqrt{2}P_2^3P_3 + 12P_1^2P_3^2 + 12P_2^2P_3^2 + 2P_3^4\big) + \frac{1}{12}\alpha_{12}\big(3P_1^4 + 6P_1^2P_2^2 + 3P_2^4 - 12\sqrt{2}P_1^2P_2P_3 + 4\sqrt{2}P_2^3P_3 + 4P_3^4\big) + \frac{1}{36}\alpha_{111}\big(9P_1^6 + 45P_1^4P_2^2 + 15P_1^2P_2^4 + 11P_2^6 + 90\sqrt{2}P_1^4P_2P_3 + 60\sqrt{2}P_1^2P_2^3P_3 - 30\sqrt{2}P_2^5P_3 + 90P_1^4P_2^2 + 180P_1^2P_2^2P_3^2 + 90P_2^4P_3^2 + 120\sqrt{2}P_1^2P_2P_3^3 - 40\sqrt{2}P_2^3P_3^3 + 60P_1^2P_3^4 + 60P_2^2P_3^4 + 4P_3^6\big) + \frac{1}{36}\alpha_{112}(9P_1^6 + 9P_1^4P_2^2 + 39P_1^2P_2^4 + 7P_2^6 - 18\sqrt{2}P_1^4P_2P_3 - 12\sqrt{2}P_1^2P_2^3P_3 + 6\sqrt{2}P_2^5P_3 - 48\sqrt{2}P_1^2P_2P_3^3 + 16\sqrt{2}P_2^3P_3^3 + 24P_1^2P_3^4 + 24P_2^2P_3^4 + 8P_3^6) + \frac{1}{108}\alpha_{123}(18P_1^4P_2^2 - 12P_1^2P_2^4 + 2P_2^6 - 18\sqrt{2}P_1^4P_2P_3 - 12\sqrt{2}P_1^2P_2^3P_3 + 6\sqrt{2}P_2^5P_3 + 9P_1^4P_2^2 + 18P_1^2P_2^2P_3^2 + 9P_2^4P_3^2 + 12\sqrt{2}P_1^2P_2P_3^3 - 4\sqrt{2}P_2^3P_3^3 - 12P_1^2P_3^4 - 12P_2^2P_3^4 + 4P_3^6) + \frac{1}{216}\alpha_{1111}\big(27P_1^8 + 252P_1^6P_2^2 + 210P_1^4P_2^4 + 28P_1^2P_2^6 + 43P_2^8 + 504\sqrt{2}P_1^6P_2P_3 + 840\sqrt{2}P_1^4P_2^3P_3 + 168\sqrt{2}P_1^2P_2^5P_3 - 168\sqrt{2}P_2^7P_3 + 504P_1^6P_3^2 + 2520P_1^4P_2^2P_3^2 + 840P_1^2P_2^4P_3^2 + 616P_2^6P_3^2 + 1680\sqrt{2}P_1^4P_2P_3^3 + 1120\sqrt{2}P_1^2P_2^3P_3^3 - 560\sqrt{2}P_2^5P_3^3 + 840P_1^4P_3^4 + 1680P_1^2P_2^2P_3^4 + 840P_2^4P_3^4 + 672\sqrt{2}P_1^2P_2P_3^5 - 224\sqrt{2}P_2^3P_3^5 + 224P_1^2P_3^6 + 224P_2^2P_3^6 + 8P_3^8\big) + \frac{1}{216}\alpha_{1112}(27P_1^8 + 72P_1^6P_2^2 + 150P_1^4P_2^4 + 128P_1^2P_2^6 + 23P_2^8 + 36\sqrt{2}P_1^6P_2P_3 + 60\sqrt{2}P_1^4P_2^3P_3 + 12\sqrt{2}P_1^2P_2^5P_3 - 12\sqrt{2}P_2^7P_3 + 90P_1^6P_3^2 - 630P_1^4P_2^2P_3^2 + 870P_1^2P_2^4P_3^2 - 10P_2^6P_3^2 - 420\sqrt{2}P_1^4P_2P_3^3 - 280\sqrt{2}P_1^2P_2^3P_3^3 + 140\sqrt{2}P_2^5P_3^3 + 60P_1^4P_3^4 + 120P_1^2P_2^2P_3^4 + 60P_2^4P_3^4 + 48\sqrt{2}P_1^2P_2P_3^5 - 16\sqrt{2}P_2^3P_3^5 + 160P_1^2P_3^6 + 160P_2^2P_3^6 + 16P_3^8) + \frac{1}{432}\alpha_{1122}\big(27P_1^8 - 36P_1^6P_2^2 + 114P_1^4P_2^4 + 188P_1^2P_2^6 + 11P_2^8 - 72\sqrt{2}P_1^6P_2P_3 - 120\sqrt{2}P_1^4P_2^3P_3 - 24\sqrt{2}P_1^2P_2^5P_3 + 24\sqrt{2}P_2^7P_3 - 72P_1^6P_3^2 + 504P_1^4P_2^2P_3^2 - 696P_1^2P_2^4P_3^2 + 8P_2^6P_3^2 + 48\sqrt{2}P_1^4P_2P_3^3 + 32\sqrt{2}P_1^2P_2^3P_3^3 - 16\sqrt{2}P_2^5P_3^3 + 96P_1^4P_3^4 + 192P_1^2P_2^2P_3^4 + 96P_2^4P_3^4 - 384\sqrt{2}P_1^2P_2P_3^5 - 128\sqrt{2}P_2^3P_3^5 + 64P_1^2P_3^6 + 64P_2^2P_3^6 + 16P_3^8\big) + \frac{1}{108}\alpha_{1123}\big(18P_1^6P_2^2 + 6P_1^4P_2^4 - 10P_1^2P_2^6 + 2P_2^8 - 18\sqrt{2}P_1^6P_2P_3 - 30\sqrt{2}P_1^4P_2^3P_3 - 6\sqrt{2}P_1^2P_2^5P_3 + 6\sqrt{2}P_2^7P_3 + 9P_1^6P_3^2 + 45P_1^4P_2^2P_3^2 + 15P_1^2P_2^4P_3^2 + 11P_2^6P_3^2 - 6\sqrt{2}P_1^4P_2P_3^3 - 4\sqrt{2}P_1^2P_2^3P_3^3 + 2\sqrt{2}P_2^5P_3^3 - 3P_1^4P_3^4 - 6P_1^2P_2^2P_3^4 - 3P_2^4P_3^4 + 12\sqrt{2}P_1^2P_2P_3^5 - 4\sqrt{2}P_2^3P_3^5 - 8P_1^2P_3^6 - 8P_2^2P_3^6 + 4P_3^8\big) - P_1E_1 - P_2E_2 - P_3E_3 \quad \text{(S3)}$$
